# Investigation of the Influence of Infrared Illumination on the Pulse Shapes of Output Signals of CdZnTe Detectors

**DOI:** 10.3390/s23249863

**Published:** 2023-12-16

**Authors:** Victor Ivanov, Viktors Fjodorovs, Sergejs Hinoverovs, Anatoli Loutchanski, Vadims Ogorodniks, Sergejs Vidinejevs

**Affiliations:** ZRF Ritec SIA, Gustava Zemgala Av. 71A, LV-1039 Riga, Latvia

**Keywords:** CdZnTe detector, quasi-hemispherical detector, sub-bandgap illumination, infrared (IR) illumination, charge collection efficiency, space charge, electric field

## Abstract

The spectrometric characteristics of CdZnTe detectors are largely determined by the nonuniformity of the material and the influence of the negative polarization effects associated with the formation of space charges in the sensitive volume of the detector. They change the electric field distribution in the detector and affect the efficiency of the charge carrier collection. An analysis of the waveforms of the output pulses was used to investigate the uniformity of the charge collection and electric field distribution in the detectors when irradiated by the alpha particles. The influence of infrared (IR) illumination on these parameters was evaluated. IR illumination had no positive effect on the planar detector but greatly improved the charge collection in quasi-hemispherical detectors in the peripheral (corner) regions. The output pulse amplitude increased, and the rise time notably decreased. Polarization that occurred predominantly in the corner regions at low temperatures (from −30 °C to −20 °C) was eliminated using IR illumination.

## 1. Introduction

X- and gamma-ray detectors based on high-resistivity CZT material are widely used in medicine, nondestructive inspection, scientific research, spectroscopy, X- and gamma-ray imaging and safeguards. Homogeneous CZT crystals with low defect density and high charge transport characteristics with electron mobility–lifetime products greater than 10^−2^ cm^2^/V [1] are currently available on the market.

Different types of CZT detectors with different contact configurations [2,3,4,5,6,7,8,9] are used to achieve the most complete charge collection and high-energy resolution. Among all electrode configurations, the quasi-hemispherical configuration [2,10,11,12,13,14,15] provides spectrometric characteristics of detectors that are sufficiently high for many tasks and do not require complex and expensive electronics for their use.

Temperature [16,17,18], high radiation fluxes [19,20,21], above-bandgap [22,23] and sub-bandgap [24,25,26,27,28,29,30,31,32,33,34,35,36] illuminations and other external influences can have both negative and positive effects on the performance of the detectors. Our investigations are focused on the influence of sub-bandgap near-infrared illumination (IR) on the performance of quasi-hemispherical detectors.

In recent years, various works have appeared with the results of studies on the different effects of sub-bandgap illumination of the near-IR range on the characteristics of CZT detectors with assumptions about their possible use. The influence of IR illumination on trapping and de-trapping processes, transport properties of charge carriers, changes in the bulk resistivity, distribution of internal electric fields, polarization and depolarization processes, current transport mechanisms, high-flux responses, space charge distributions and charge collections has been studied.

However, there are few works describing the effect of IR illumination on the spectrometric properties of detectors, namely, on energy resolution in the literature.

In [26], an improvement in the spectrometric characteristics of a planar CZT detector with dimensions of 5 × 5 × 1 mm under IR illumination at a wavelength of 940 nm was obtained when recording radiation from a ^241^Am source. The energy resolution of the 59.6 keV line improved from 9.71% to 5.68%. The improvement was attributed to the effect of illumination on charge carrier collection by suppressing trapping and encouraging de-trapping of the trapped carriers.

In [27], in a capacitive Frisch-grid detector with dimensions of 5 × 5 × 8 mm at the ^137^Cs 662 keV line under IR illumination at a wavelength of 940 nm, an improvement in the energy resolution was obtained from 3.3% to 2.3%.

A slight improvement in the energy resolution (from 4.21% to 3.18%) of the ^137^Cs 662 keV line under IR illumination at a wavelength of 940 nm in a coplanar grid detector with dimensions of 10 × 10 × 10 mm was reported in [28].

Improvements in the spectrometric characteristics of quasi-hemispherical detectors were reported in [25,29,36,37].

In [29], using a quasi-hemispherical CZT detector (Savannah River National Laboratory, Aiken, SC, USA, and Fisk University, Nashville, TN, USA) with dimensions of 9.93 × 10 × 4.5 mm, the energy resolution at 662 keV of 137Cs was obtained as 3.64% without IR illumination and 2.49% when illuminated at a wavelength of 950 nm. Improvement in energy resolution, but to a lesser extent, was also obtained when IR illumination was at a wavelength of 1000 nm.

In [37], the possibility of significant improvement and stabilization of the spectrometric characteristics of quasi-hemispherical detectors operating under various conditions with IR stimulation was shown. Thus, when a detector of 4000 mm^3^ volume was exposed to IR illumination with a wavelength of 940 nm, the energy resolutions of 2.5% and 1.3% at 662 keV line were obtained at room temperature and +5 °C, respectively.

The results obtained from studies of the performance of spectrometric quasi-hemispherical detectors under the influence of IR illumination differ significantly from those obtained in other studies with other detectors. The purpose of this research is to determine the characteristics of quasi-hemispherical detectors that can achieve significant improvements in performance, at both room temperature and low temperatures.

To study the processes of charge carrier collection in quasi-hemispherical detectors, the transient current or transient charge methods [38,39,40] were used. These methods are based on measuring and analyzing waveforms of current or voltage pulses induced by generated charges as they move in the electric field of the detector. Any changes in the distribution of the electric field in the detector are accompanied by a change in the waveforms of the recorded pulses. In our study, we used the transient charge or voltage method. To generate one type of charge carrier, we used alpha particles with a shallow penetration depth into the detector [41,42,43].

## 2. Experiment

To fabricate detectors in this study, CZT crystals were grown using the traveling heater method (THM) in Redlen Technologies (Victoria, BC, Canada). The (μτ)_e_ product for electrons of the used CZT samples was no lower than 10^−2^ cm^2^/V.

In our study, several planar detectors were used in the shape of a rectangular parallelepiped: two with dimensions of 20 × 20 mm and a thickness of 10 mm and two with dimensions of 15 × 15 mm and a thickness of 7.5 mm. The detectors contained electrodes on two large sides.

After all tests with the planar detectors, the gold contacts were removed, the crystals were subjected to repeated mechanical and chemical polishing, and quasi-hemispherical detectors with dimensions of 20 × 20 ×10 mm and 15 × 15 × 7.5 mm were made from the same crystals. They contained a large negative electrode on five sides and a positive dot electrode with a diameter of 2 mm at the center of one of the large sides. This optimal diameter of the dot electrode was determined by the size of the detector and the electrophysical characteristics of the CZT crystal used [14].

All detectors were produced with the same surface treatment. The crystals were mechanically and chemically polished to remove surface mechanical damage. Electrochemically deposited gold contacts from a solution of 1% hydrochloroaurate acid were used for both planar and quasi-hemispherical detectors.

In our experiments, we used the transient charge technique. In this method, the voltage or charge pulses induced at the detector electrodes were recorded for further processing. Alpha particles with an energy of 5.5 MeV emitted by ^238^Pu were used for charge generation. To ensure single-charge collection conditions [43] due to electron collection, detectors were irradiated from the cathode side in all experiments. We analyzed only pulses caused by electron collection since fast electronic components mainly determine the quality of the detector during single-charge collection.

**Figure 1:**
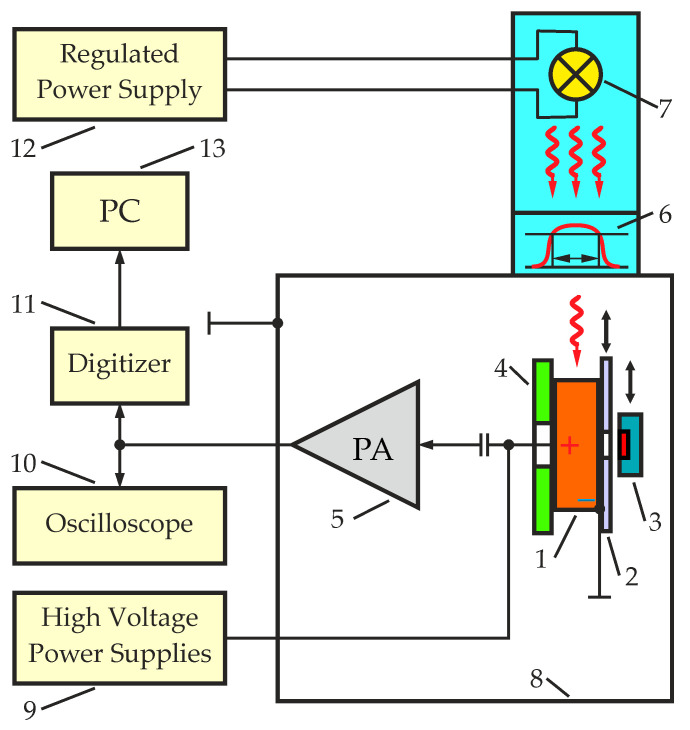
Schematic of the measuring setup: 1—CZT detector, 2—aluminum foil collimator, 3—alpha-particle source, 4—detector holder, 5—charge-sensitive preamplifier (PA), 6—bandpass filter, 7—halogen lamp, 8—lightproof and electrically shielded case, 9—high-voltage power supply, 10—digital oscilloscope, 11—digitizer, 12—regulated power supply, 13—personal computer (PC).

Figure 1 shows a block schematic of an experimental measuring setup used for the measurements. The CZT detector (1) was placed on a holder (4) located in a lightproof and electrically shielded casing of the measuring setup (8); this setup allowed alternate placement of planar or quasi-hemispherical detectors.

The alpha-particle source (3) (Figure 1) was located as close as possible to the detector surface at a distance of 5 mm in the measuring setup. Since the measuring setup was nonvacuum, the energy loss of alpha particles in the air had to be reduced during measurements. To provide alpha irradiation of the detector at different regions on the surface, an aluminum foil collimator (2) with a diameter of 2 mm was used. The collimator was alternately attached to the surface of the detector at different measurement regions. The alpha-particle source was mounted on a movable holder, which made it possible to place the source exactly above the collimator hole to ensure a normal incidence of alpha particles on the detector surface. Thus, the energy loss of alpha particles in the input window of the detector due to the different angles of incidence on the detector surface was also reduced.

Signals from a charge-sensitive preamplifier (5) (Figure 1) output were directly fed to the input of a digitizer without any preprocessing. This eliminated a possible influence of the shaping circuit on the amplitudes and shapes of the output signals. Parameters of the output signals were controlled using a Siglent digital (Siglent Technologies Co., Ltd., Shenzhen, China) oscilloscope model SDC 2104X (10). To digitize output signals, a Teledyne SP Devices (Teledyne SP Devices, Linköping, Sweden) digitizer model ADQ14DC-4C-PCIE (11) with a sample rate of 1 Gsps and 14-bit vertical resolution was used. The detector was powered by an ISEG high-voltage power supply (iseg Spezialelektronik GmbH, Radeberg, Germany) model SHR 2260 (9).

The IR illumination of the detector was carried out with the use of a halogen lamp (7) (Figure 1) and a Thorlabs Inc. (Thorlabs Sweden AB, Mölndal, Sweden) bandpass filter with central wavelengths of 1050 nm (FBH1050-10) or 940 nm (FBH940-10) (6). The full peak width at half maximum (FWHM) of the transmission spectra of the filters used was 10 nm. The filters had diameters of 25 mm, providing uniform illumination of the detectors. A regulated power supply (12) made it possible to easily adjust the illumination intensity of the halogen lamp.

**Figure 2:**
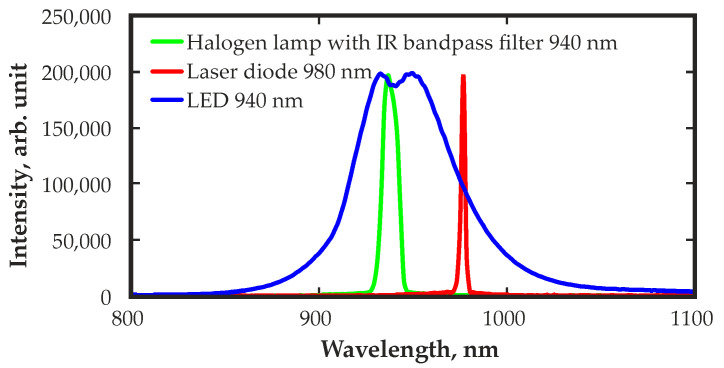
Emission spectra of the IR LED, IR laser diode and transmission spectrum of the IR bandpass filter.

To evaluate the feasibility and ease of use, other types of IR illumination sources were also investigated to improve the spectrometric performance of CZT detectors. Among them were Roither Lasertechnik GmbH (Roithner Lasertechnik GmbH, Vienna, Austria) surface-mounted IR light emission diode (LED) SMC940 (peak wavelength of 940 ± 10 nm, FWHM = 50 nm) and IR laser diode LDM-0980-050m-50 (peak wavelength of 975 ± 15 nm, FWHM < 3.0 nm). For comparison, Figure 2 shows the emission spectra of the above-mentioned IR sources as well as the IR bandpass transmission spectrum. All spectra were obtained using a SARSPEC NIR spectrometer model ProNIR 1 (Sarspec Lda., Porto, Portugal).

Signals from the digitizer (11) (Figure 1) were transmitted to a PC for further processing. The waveforms of the output pulses, amplitude-to-time distributions, amplitude spectra and transit time distributions were obtained. Analysis of these data and their changes under IR illumination was used to evaluate the process of charge collection (degree of uniformity and efficiency across the detector) and changes in the electric field distribution in the detector.

Measurements in a temperature range from −30 °C to +50 °C were carried out in a SANWOOD temperature test chamber model SM-22-CC. A special sealed measuring fixture with the installed quasi-hemispherical detector with dimensions of 15 × 15 × 7.5 mm, the charge-sensitive preamplifier and the source of alpha particles inside were in the chamber. Around the detector, several IR LEDs with two wavelengths of emitted light (1050 nm and 940 nm) were arranged. Several IR LEDs of the same type and wavelength of emitted light were arranged to ensure more uniform illumination of the detector. The registration of alpha particles was carried out as described earlier (collimation, alpha source location and pulse processing). The intensity of the IR illumination was adjusted by changing the forward current of the selected IR LEDs using a current source. The maximum permissible forward current of the diodes used was 50 mA.

A measuring setup for scanning the surface of the initial planar detectors with alpha particles was used to determine the mobility and the mobility–lifetime product of electrons (µτ)_e_ and to assess the degree of homogeneity of the initial CZT crystals intended for quasi-hemispherical detector fabrication later. When measuring alpha spectra, the planar detector was placed on the holder with the cathode contact down. The holder had a wide window that left almost the entire surface of the detector open for alpha particles to enter. The alpha source was located above the detector cathode at 1 mm.

A GBS-Elektronik GmbH (GBS Elektronik GmbH, Radeberg, Germany) digital spectrometer MCA527 was used to record the amplitude spectra of alpha particles, as well as to supply low-voltage power to the preamplifier and bias voltage to the detector. During the measurements, to eliminate possible charge losses in the amplitude of the recorded signals due to the ballistic effect [44], the shaping and flattop times of the digital amplifier were selected taking into account the maximum transit time of charge carriers in a 1 cm thick detector, which was approximately 2 μs at a bias voltage of 500 V.

All CZT samples used for the experiments underwent incoming inspection. The samples were inspected using IR transmission microscopy based on application of a BMS Microscopes (BMS Microscopes b.v., Capelle aan den IJssel, Netherlands) IR digital CMOS camera ProfCam 5.1 Mpixel. The presence, size and uniformity of the distribution of inhomogeneities in the samples were assessed. The use of IR microscopy is a traditional way to monitor inhomogeneities in CZT detectors.

## 3. Results and Discussion

### 3.1. Planar Detector

To estimate the homogeneity of the CZT used to fabricate the detectors, all samples were inspected using IR transmission microscopy at 940 nm near-infrared light. Inclusions and structural inhomogeneities of the material, their sizes and the uniformity of distribution over the volume were of interest. Small tellurium inclusions no larger than 8 microns in size were randomly distributed throughout the entire volume. The density of the observed inclusions was approximately 10^5^ cm^−3^. There were no large inclusions or accumulations of small inhomogeneities along the grain boundaries. Thus, in transmitted near-infrared light, the material inspected showed acceptable homogeneity.

After the inspection, planar detectors with continuous contacts on two opposite large faces of the sample were manufactured. The amplitude spectra obtained during the detection of alpha particles were used to estimate the value of the mobility–lifetime product of electrons (µτ)_e_. when scanning different regions of the detector surface with the alpha source. To determine (µτ)_e_ values with the traditional approach using the Hecht equation [45] usually necessary to plot the dependence of the position of the recorded alpha peaks on the bias voltage and subsequently fit the parameters to the equation. In our work, a simplified approach was used when alpha spectra were measured at two bias voltages of 500 and 1000 V at each point. Then, the positions of the maxima of the recorded alpha peaks in the channels were determined. The value of the (µτ)_e_ product was calculated using the formula obtained from the Hecht equation:

(μτ)_e_ = d^2^/(2·U_1_·ln(A_2_/(2 A_1_ − A_2_))) (1)

where d is detector thickness, cm; A_1_ is the position of the alpha-peak maximum in the channels measured at bias voltage U_1_; and A_2_ is the position of the alpha-peak maximum in the channels measured at bias voltage U_2_ with U_2_ = 2·U_1_.

It is noteworthy that this calculation is based on the application of the Hecht equation, which is applicable only when the electric field and the traps are uniformly distributed throughout the detector volume.

**Figure 3:**
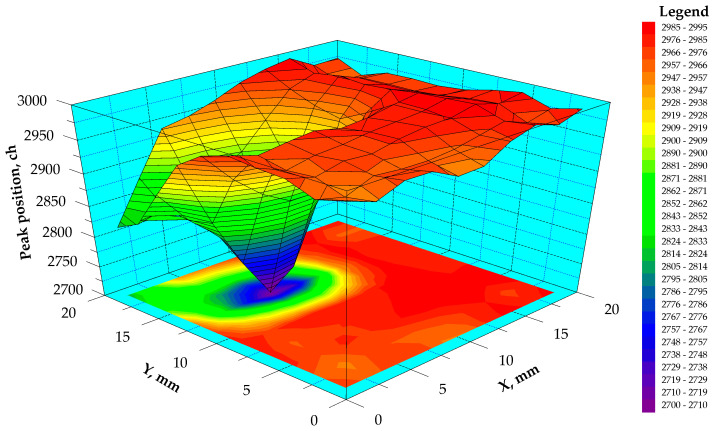
Three-dimensional surface distribution map of the alpha-peak position.

The result of the surface scanning of one of the planar detectors with dimensions of 20 × 20 × 10 mm is shown in Figure 3. In one region of the detector surface, a noticeable decrease in the signal amplitude was observed. This could be caused by a decrease in the efficiency of charge carrier collection from this region or by some inhomogeneity in the thickness of the gold contact layer. An increase in the thickness of the gold layer can lead to an additional loss of alpha-particle energy at the input contact of the detector and a decrease in the amplitudes of the recorded pulses. However, remaking the contact after its complete removal did not eliminate the resulting decrease in amplitude in the indicated region. This confirmed the assumption that the decrease in amplitude is associated with a decrease in the efficiency of charge carrier collection from this region. Further research also confirmed the correctness of this assumption. Measurements with an IR microscope did not show the presence of any inhomogeneities in this region.

The calculations using Equation (1) resulted in an average value of (µτ)_e_ = (1.6 ± 0.3) × 10^–2^ cm^2^/V for all scanned regions of the detector, excluding the region with reduced amplitude where (µτ)_e_ = 2.2 × 10^−3^ cm^2^/V was significantly lower. This could indicate a significant distortion of the electric field distribution in this region. The distortion could arise owing to the presence of any structural disturbances in the detector that are not visible under an IR transmission microscope. For example, this could be caused by star-like defects formed after post-growth annealing [46]. It was also reported in [47] that the Redlen CZT material can possess “vestiges” (crystalline deformation) of the secondary phase after post-growth annealing, which are not visible in IR transmission microscopy.

The scanning of the surfaces of other investigated planar detectors did not show the presence of any noticeable nonuniformities. The calculation results for the (μτ)_e_ products were no lower than 10^–2^ cm^2^/V.

The detector with the indicated nonuniformity was placed in a measuring setup (Figure 1) for more detailed research. Transient voltage pulses obtained by irradiating a selected region of the detector surface with alpha particles were digitized and saved in a List Mode in a single file for each selected region. In total, data from 13 different regions on the detector surface were recorded. For each single pulse, the amplitude and transit time were determined.

The transit time was determined from the rise time of the output voltage pulse based on the matching of 10% to 90% of the output pulse rising edge. This is a standard, frequently used rise time evaluation approach.

The rise time was determined by the magnitude of the bias voltage applied to the detector and was very sensitive to the distribution of the electric field in the detector.

**Figure 4:**
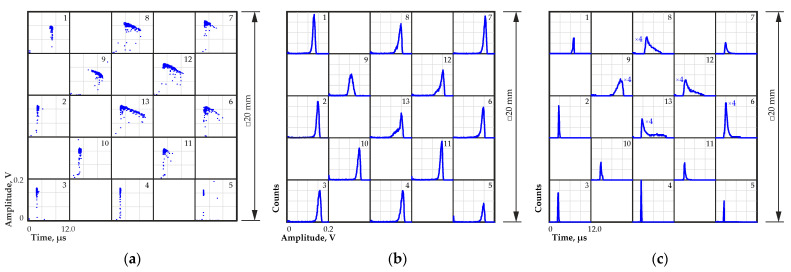
Amplitude to transit time distributions (**a**) and pulse amplitude spectra (**b**) and transit time distributions (**c**) for different regions on the detector surface. Numbers from 1 to 13 indicate the number of a region on the detector surface selected for measurement.

After data processing, the amplitude to transit time distributions were constructed for different regions on the detector surface (Figure 4a). Measurements were carried out at a bias voltage of 800 V.

Using the obtained amplitude to transit time distributions, the pulse amplitude spectra and transit time distributions were calculated. Figure 4 shows the shapes of the calculated amplitude spectra (b) and transit time distributions (c) for all the chosen irradiated regions on the detector surface. The spectra and distributions presented in Figure 4b,c and similar ones are illustrative; they only display shape, amplitude and time values. The number of pulses in the spectra and distributions does not reflect the actual ratio of the number of pulses received from each region.

Large variations in amplitude and transit time were observed on part of the detector surface. The longest transit time and smaller amplitude were observed in regions 1 and 9. Regions 8, 12 and 13 had the widest distributions of transit time. In these transit time distributions, along with relatively short transit times corresponding to the maximum of the distribution, there is a certain number of longer transit times. The location of these regions corresponded to the detected region with decreased charge collection efficiency obtained when scanning the detector surface with alpha particles in the previous experiment (Figure 3). The large width of the distributions in regions 8, 12 and 13 could be due to the relatively large diameter of the collimator, which covered several regions with different transit times.

Data on the positions of the maxima of the amplitude spectra and the maxima of the transit time distributions for all tested regions on the detector surface are presented in Table 1.

**Figure 5:**
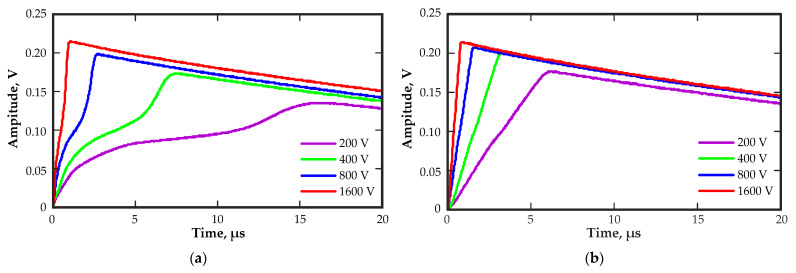
Output pulse waveforms from regions 9 (**a**) and 5 (**b**) at different bias voltages.

A significant difference in the rising edge shapes of the recorded output pulses was obtained. Figure 5 shows the typical output pulse waveforms from regions 9 and 5 at different bias voltages. The close to the linear rising edge of the output pulses from region 5 indicated the presence of a uniform distribution of the electric field in this region. The rising edge of the output pulses from region 9 had a complex shape. The signal first increased quickly, then slowed down and then increased rapidly again. This waveform of the output signal could indicate a highly nonuniform electric field in this region of the detector. The electric field had an increased field strength near the cathode and anode. The change in the waveforms of the output pulses on the value of the bias voltage is visible.

Notably, increasing the bias voltage decreased the rise time and increased the signal amplitude for pulses from both regions. However, the shape of the rising edge of the pulses from region 9 remained complex.

**Figure 6:**
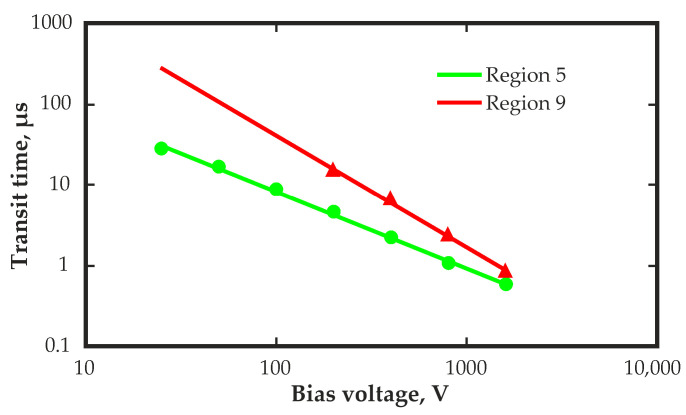
The double-logarithmic dependence of the transit time measured on the bias voltage for two regions on the detector surface.

In Figure 6, a double logarithmic scale shows the dependence of the transit time measured on the bias voltage for two regions of the surface of the detector.

The dependence for region 5 is close to inversely proportional, which is typical for a uniform distribution of the electric field.

Using data on transit times T_e_ (s), the electron mobility (μ)_e_ was calculated at various voltages U (V), using the well-known carrier mobility expression applicable to a uniform electric field distribution:

(μ)_e_ = d^2^/(U·T_e_) (2)

where d is detector thickness, cm, and U is bias voltage, V.

The mobility calculated for region 5 was approximately 1108 ± 50 cm^2^·V^–1^·s^–1^ for all bias voltages. The mobility calculated for region 9 was significantly smaller and increased from 330 to 740 cm^2^·V^–1^·s^–1^ as the bias voltage increased from 200 V to 1600 V. This result may indicate some incorrectness in the application of Equation (2) to a detector with a nonuniform electric field distribution.

Using data on the position of the alpha-peak maxima when the bias voltage was doubled, the parameter (μτ)_e_ was also calculated employing Equation (1). Calculations at different bias voltages resulted in (μτ)_e_ equal to (1.15 ± 0.2) × 10^–2^ and (3.2–5.5) × 10^–3^ cm^2^/V for regions 5 and 9, respectively. In the latter case, the different values obtained indicate that the application of Equation (1) is incorrect if the electric field is distributed nonuniformly.

**Figure 7:**
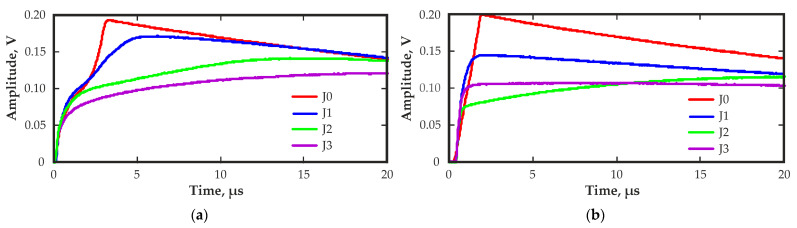
Output pulse waveforms from region 9 (**a**) and region 5 (**b**) measured without (J0) and with IR illumination at 940 nm at different illumination levels (J1 < J2 < J3).

The effect of IR illumination on the waveforms of the output pulses received by the planar detector was studied. Figure 7 shows the change in the waveform of the output pulses from regions 9 and 5 at different levels of IR illumination at a wavelength of 940 nm.

During measurements, IR light entered the detector through a side surface that had no contacts. The illumination did not improve charge collection in either poor region 9 or good region 5 at low illumination levels and even worsened at high levels. In general, IR illumination affected the duration and shape of the rising edge of the output pulses. This occurred owing to a small increase in the electric field strength near the cathode at low illumination levels and a large increase in the electric field strength near the cathode, as well as a drop in the field strength in the rest of the detector at high illumination levels. These changes in the electric field distribution led to a decrease in the output pulse amplitudes and a significant increase in the duration of the transit times. An increase in the electric field strength near the cathode may be associated with the formation of a positive space charge due to the excitation of holes under the influence of IR illumination. Similar behavior of CZT detectors under the influence of IR light has been described in various publications [22,29,30].

### 3.2. Quasi-Hemispherical Detector

The fabricated quasi-hemispherical detector was placed in the measuring setup (Figure 1), and transient voltage pulses were obtained and saved using the same method applied to the planar detector. Alpha particles irradiated the detector from the side of the larger negative contact. The numbering and location of the regions on the surface of the detector were the same as in the measurements of the original planar detector.

Planar detectors, as a rule, have a uniformly distributed electric field both over the area and across the thickness of the detector. Hemispherical detectors ideally have an electric field corresponding to that of a spherical capacitor. This, as well as a small pixel effect [48], allows the realization of single-charge collection. Quasi-hemispherical detectors have additional field inhomogeneity in corner regions, which significantly affects their characteristics. Accordingly, when scanning a surface, the efficiency of collecting charge carriers from different regions is approximately the same for a planar detector (without structural inhomogeneities) and is significantly inhomogeneous for a quasi-hemispherical detector.

**Figure 8:**
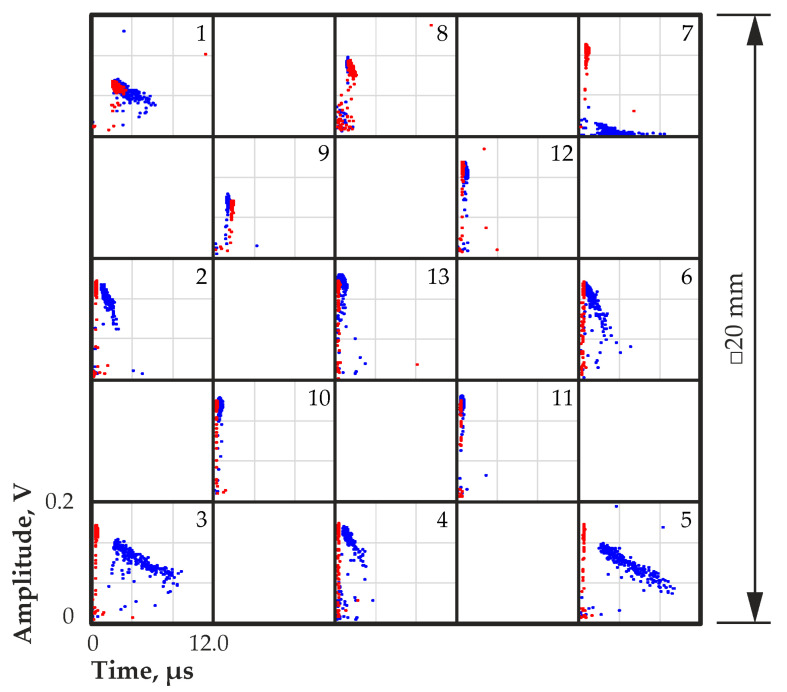
Amplitude to transit time distributions for different regions on the quasi-hemispherical detector surface with (red dot) and without (blue dot) IR illumination at a wavelength of 940 nm. Numbers from 1 to 13 indicate the number of a region on the detector surface selected for measurement.

**Figure 9:**
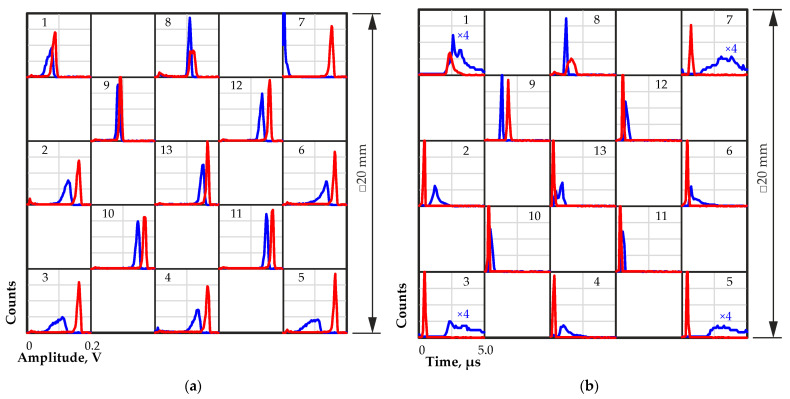
Pulse amplitude spectra (**a**) and transit time distributions (**b**) for different regions on the quasi-hemispherical detector surface with (red line) and without (blue line) IR illumination at a wavelength of 940 nm. Measurements were performed using a quasi-hemispherical detector with dimensions of 20 × 20 × 10 mm at a bias voltage of 800 V. Numbers from 1 to 13 indicate the number of a region on the detector surface selected for measurement.

Figure 8 shows the amplitude to transit time distributions obtained from different regions on the quasi-hemispherical detector surface at a bias voltage of 800 V. From these data, the pulse amplitude spectra and the transit time distributions were calculated, as shown in Figure 9.

Along the surface of the detector, the greatest change in amplitude and transit time was observed in the peripheral, predominantly corner regions. The narrow peaks in the amplitude spectra in regions 10–13 with a large amplitude indicate a more uniform and more complete charge collection compared with corner regions 1, 3, 5 and 7 (Figure 9).

Data on the positions of the maxima of the amplitude spectra and the maxima of the transit time distributions for all tested regions on the detector surface with and without IR illumination are presented in Table 2.

**Figure 10:**
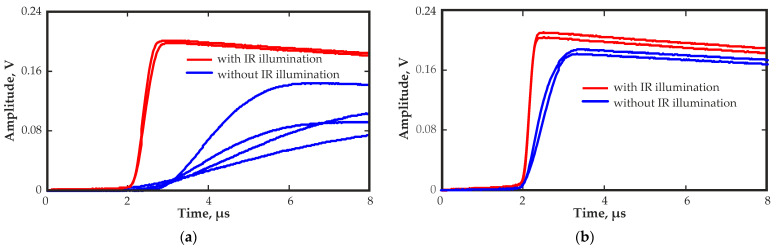
Waveforms of the output pulse from corner region 7 (**a**) and central region 13 (**b**) of the quasi-hemispherical detector with and without IR illumination at a wavelength of 940 nm.

At the corners, a significant decrease in the amplitude, a broadening of the peaks in the amplitude spectra, and an increase in the transit time were observed. This could be due to a significant decrease in the electric field strength. The calculation results in [14] showed that in the corner regions of quasi-hemispherical detectors, the electric field can be significantly weakened, which confirms our assumption. An increase in the transit time led to an increase in charge losses due to an increase in the probability of the charge carrier trapping. Charge collection in the central regions of the detector was much more uniform, and the transit times were much faster than in the corner regions. Figure 10 shows the waveforms of the output pulses of corner region 7 (a) and central region 13 (b).

In addition to poor charge collection from the peripheral regions of the detector under study, there is another region 9, with a noticeably reduced pulse amplitude and increased transit time. The location of this region corresponded to the region with decreased charge collection efficiency found when scanning the surface of the original planar detector with alpha particles in the previous experiments.

The effect of IR illumination on the characteristics of the output pulses of the quasi-hemispherical detector obtained under alpha-particle irradiation was investigated. As shown in [36], IR illumination has a significant impact on the characteristics of quasi-hemispherical detectors. We studied in more detail the processes occurring in quasi-hemispherical detectors under the influence of IR radiation to clarify the features of charge-carrier collection in them in comparison with CZT detectors of other types. This will further help optimize the performance of quasi-hemispherical detectors under various operating conditions.

IR illumination was carried out from a side surface of the quasi-hemispherical detector with a gold contact. Noticeably, a metal contact layer reduces the intensity of the IR radiation penetrating into the detector. Thus, a gold contact can reduce the transmission of IR illumination by hundreds of times depending on the thickness of the contact [36]. Nevertheless, IR illumination improved charge collection from the corner regions. A comparison of the waveforms of the output pulses from corner region 7 and central region 13 with IR illumination at a wavelength of 940 nm confirmed this (Figure 10). The transit time of the output pulses was significantly reduced, and the amplitude increased in the corner region owing to an increase in the electric field strength there. The pulse amplitude from the central region 13 increased slightly, and the pulse rise times decreased slightly.

Figure 8 and Figure 9 show the amplitude to transit time distributions and the amplitude spectra and transit time distributions, respectively, obtained from different regions on the surface of the detector with IR illumination at a wavelength of 940 nm. A significant improvement in charge collection was obvious over almost the entire detector surface. An increase in the output pulse amplitudes and a decrease in the transit times were observed, especially for the peripheral regions of the detector. This was due to an increase in the electric field strength in these regions caused by the redistribution of the space charge in the detector under the influence of IR illumination.

Improvement in charge collection under IR illumination in region 9 was not observed, and only a minor improvement in charge collection in regions 1 and 8 adjacent to region 9 was observed.

The pulse amplitudes in these regions increased slightly, as did the transit times in regions 9 and 8. As already noted, these regions coincided with the same region on the surface of the original planar detector where the deterioration in charge collection was also observed. No noticeable changes in the output pulse waveforms under the impact of IR illumination were observed. This result confirmed the assumption of the presence of structural heterogeneity in the volume of the detector, which was not detected during IR transmission inspection. It can also be assumed that the slight increase in amplitude and increase in transit time are associated with a suboptimal illumination level for that particular region. As was shown in [37], an excessive level of IR illumination leads to a deterioration in the spectrometric properties of hemispherical detectors. Excessive IR illumination not only causes an increase in current noise but also significantly increases the transit time. The latter is determined by the formation of an extended space charge, which significantly increases the transit time.

**Figure 11:**
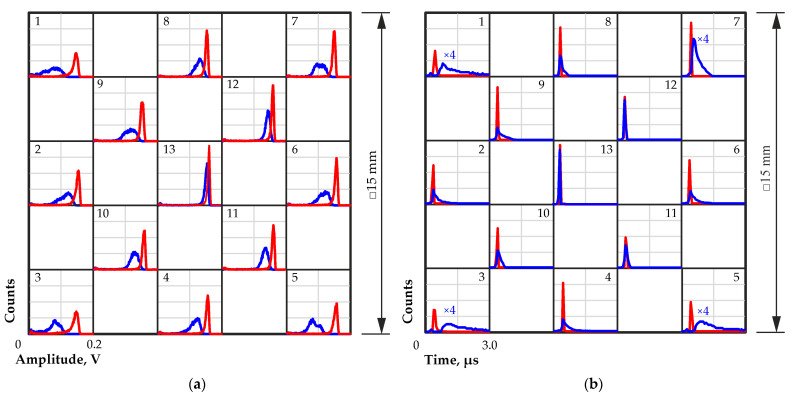
Pulse amplitude spectra (**a**) and transit time distributions (**b**) from different regions on the detector surface with (red line) and without (blue line) IR illumination at a wavelength of 940 nm. Measurements were performed using a quasi-hemispherical detector with dimensions of 15 × 15 × 7.5 mm at a bias voltage of 800 V. Numbers from 1 to 13 indicate the number of a region on the detector surface selected for measurement.

Additionally, experiments with IR illumination according to the procedure described above were carried out on two quasi-hemispherical detectors with dimensions of 15 × 15 × 7.5 mm. In Figure 11, the amplitude spectra (a) and pulse transit time distributions (b) from different regions obtained with and without IR illumination at a wavelength of 940 nm are shown.

Similar to the results obtained with a larger quasi-hemispherical detector, worsened charge carrier collection in the corner regions of the detector was observed. IR illumination led to a significant improvement in charge collection in all peripheral regions of the detector. There was also an improvement in charge collection in the central region of the detector, but it was insignificant.

Data on the positions of the maxima of the amplitude spectra and the maxima of the transit time distributions for all tested regions on the detector surface without and with IR illumination are presented in Table 3.

The transit times obtained in the peripheral regions of the detector without IR illumination were noticeably longer than those from the central region of the detector. The use of IR illumination noticeably reduced this difference, improved the shapes of the amplitude spectra and made the charge collection over the entire surface of the detector more uniform. The same improvement in performance under the influence of IR illumination was observed in all other tested quasi-hemispherical detectors.

### 3.3. Measurements over a Temperature Range

To carry out measurements over a temperature range from −30 °C to +50 °C, a special sealed fixture placed in the temperature test chamber was used. The fixture allowed alternating illumination with IR LEDs with different emission wavelengths and adjustable intensities. A quasi-hemispherical detector with dimensions of 15 × 15 × 7.5 mm was irradiated through a collimator in the corner or center with an alpha-particle source located 5 mm from the larger surface with a negative contact. The resulting transient voltage pulses were digitized and saved using the same method applied in previous experiments. The detector bias voltage was 800 V.

**Figure 12:**
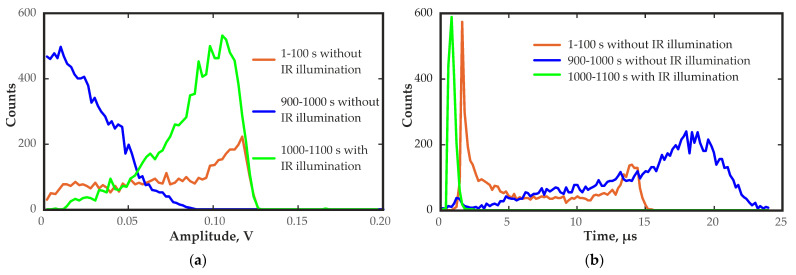
Pulse amplitude spectra (**a**) and transit time distributions (**b**) for the different time periods after the bias voltage was applied 0–100 s and 900–1000 s without and with IR illumination at a wavelength of 1050 nm 1000–1100 s.

In the corner region of the detector at the lowest temperature of −30 °C, significant changes owing to polarization were detected. The pulse amplitude spectra decreased, the calculated transit time distributions significantly increased (Figure 12), and the pulse rising edge shape changed over the operation time after the detector bias voltage was applied.

The amplitude spectra and the transit time distributions presented in Figure 12, as before, are illustrative; they only display shape, amplitude and time values. The number of pulses in the spectra and the distributions does not reflect the actual ratio of the number of pulses obtained under different conditions. The main changes in the distribution of the electric field occurred in the first tens of seconds after the detector bias voltage was applied. Within the first seconds, pulses with an average transit time of approximately 1.6–2 µs were observed. After 100 s, the average transit time was increased to approximately 14 µs (Figure 12). However, to achieve reliable statistics, a pulse-acquisition time of at least 100 s was required. Therefore, the transit time distribution calculated from the pulses recorded during the first 100 s after the bias voltage was applied already contained the transit times for the pulses with long rising edges and reduced amplitudes.

**Figure 13:**
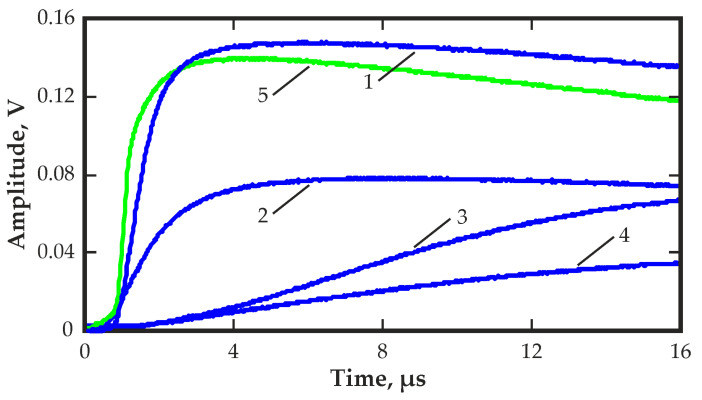
Typical waveforms of output pulses at different times after the bias voltage was applied (blue colour) for 10 (1), 100 (2), 600 (3) and 1200 s (4) and with IR illumination (green colour) at a wavelength of 1050 nm (5).

Figure 13 shows the waveforms of the output pulses at different times after the bias voltage is applied. Turning to IR illumination with a wavelength of 1050 nm changed radically the waveforms of the output pulses and improved the amplitude spectra and transit time distributions. The wavelength of 1050 nm was selected because it made it possible to significantly improve the stability of the quasi-hemispherical detectors at low temperatures, as shown in [37].

The duration of the rising edge of the pulses increased significantly over time. This may indicate a decrease in the electric field in the corner region of the detector over time. This was due to the polarization effect associated with the capture of the charge carriers by traps and the formation of a space charge.

Figure 12 shows the pulse amplitude spectrum and the transit time distribution obtained with IR illumination at a wavelength of 1050 nm. Almost complete restoration of the amplitude spectrum was observed immediately after the illumination was applied. The transit time decreased significantly approximately from 20 µs to 0.8 µs, which was even lower than the value at the first moment after applying the bias voltage. This was due to the depolarization effect of IR illumination at this wavelength, which released the trapped charge carriers, eliminated space charges and restored the electric field in the corner region of the detector. The evolution of the signal waveform determined by the change in the distribution of the electric field is clearly visible in Figure 13.

**Figure 14:**
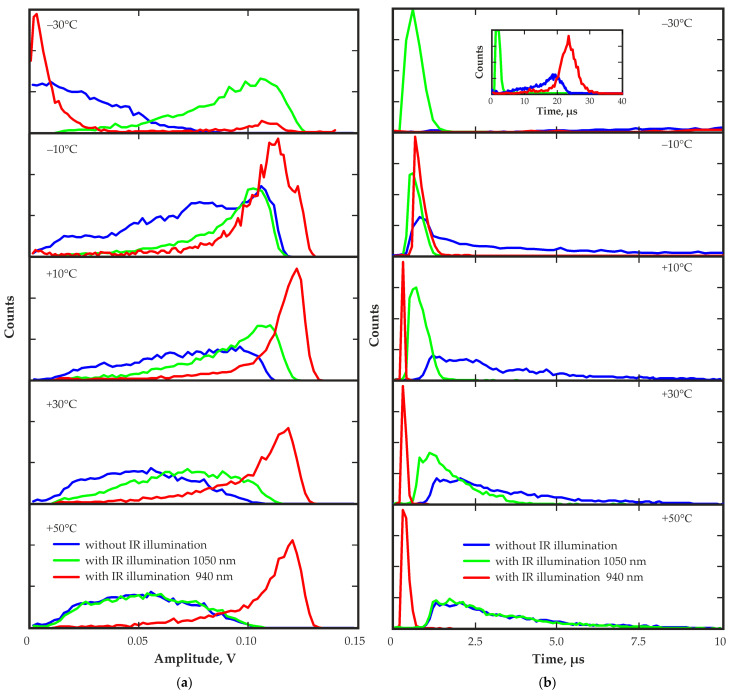
Pulse amplitude spectra (**a**) and transit time distributions (**b**) from the corner region of the detector at different operating temperatures with and without IR illumination.

Figure 14 shows the amplitude spectra and transit time distributions from the corner region of the detector at different operating temperatures with and without IR illumination at wavelengths of 940 nm or 1050 nm.

For each temperature, the detector was held under the applied bias voltage for 30 min to stabilize characteristics and identify possible polarization before starting measurements. After turning on the IR illumination, the detector was also kept for 30 min before starting measurements.

When the temperature was lowered to −10 °C without IR illumination, only a slight increase in the amplitude and a slight change in the shape of the transit time distribution were observed. Polarization, expressed as a change in the characteristics over time, was not detected in this temperature range. As mentioned above, significant polarization was observed at −30 °C.

Figure 14 also demonstrates the effect of IR illumination with different wavelengths on the amplitude spectra and the transit time distributions. IR illumination with a wavelength of 1050 nm noticeably improved charge collection at low temperatures. Amplitude spectra and transit time distributions were almost completely restored. As the temperature increased, the efficiency of this IR illumination at a wavelength of 1050 nm decreased and had practically no effect on the charge collection process at temperatures above room temperature.

In contrast, IR illumination with a wavelength of 940 nm had a positive effect at positive temperatures. The use of IR illumination significantly increased the amplitude and reduced the transit time to the maximum tested temperature of +50 °C. For each temperature, there was an optimal level of IR illumination intensity that provided the best conditions for charge collection.

Using this IR illumination at a temperature of −30 °C did not provide a positive result. After operating under bias voltage and reaching a stable state, the detector was illuminated with IR light of minimum intensity. At the first instant after turning on the IR illumination, there was a slight increase in amplitude and a decrease in transit time. Then, there was a rapid deterioration in performance. The amplitude decreased, and the transit time increased to the values before illumination. Figure 14 shows the amplitude spectrum and the transit time distribution obtained over 30 min after turning on the illumination.

A completely different behavior of the charge collection process with operation temperature changes was observed when the collimator was placed in the central region of the detector during similar measurements. No significant changes in the shape of the output pulses, changes in the amplitude spectra or the transit time distributions depending on temperature were detected. No changes in the output pulse shape over time were observed at any temperature. This result indicated the absence of visible polarization and the constancy of the electric field distribution in the investigated range of temperatures in the central region of the detector.

In the central region of the detector, no noticeable changes in the shape of the output pulses and their amplitude were obtained over the entire tested temperature range under IR illumination. The average rise time of the output pulses was observed to decrease from 1.2 µs to approximately 0.3 µs when exposed to IR illumination with a wavelength of 1050 nm at −30 °C.

In quasi-hemispherical detectors, the temperature dependences of the charge-collection process in the central and corner regions differ due to the low strength of the electric field in the corner regions. Such a low field slows down the speed of charge carriers and increases trapping probability, leading to the formation of a space charge. A high-strength field in the central region forces charge carriers to move faster. This reduces the probability of trapping and prevents the formation of space charges in the central region of the detector.

This investigation showed that the deterioration of the characteristics of quasi-hemispherical detectors and their instability at low temperatures are determined by the occurrence of polarization associated with the formation of space charges, causing a decrease in the electric field in the corner regions of the detector. The use of IR illumination at a wavelength of 1050 nm leads to the restoration of detector characteristics due to depolarization.

It is noteworthy that IR illumination was carried out using IR LEDs. The FWHM of the IR LED emission spectrum is relatively wide. The FWHM of the IR LED used is 50 nm, but the full peak width at 1/10 maximum (FWTM) of the same IR LED emission spectrum is approximately 125 nm. Such a large spectral width could lead to large inaccuracies in assessing the influence of a particular wavelength of IR illumination and its intensity on the characteristics of the detector. For example, in the emission spectrum of the IR LED used with the specified wavelength of 1050 nm, approximately 4% of the total emitted light falls in the range below 940 nm, and the short-wavelength tail in the spectrum extends to almost 850 nm. This short-wavelength IR light can have a large impact on detectors. In this case, it is incorrect to state that it is only IR light with a wavelength of 940 nm or 1050 nm specified in the LED specification that affects the characteristics of the detector.

In addition, other features of LEDs must be taken into account. The wavelength corresponding to the maximum emissivity of the LED is indicated in the manufacturer’s specification with some errors and needs to be considered. For example, for a 940 nm LED, the permissible error is ± 10 nm. Additionally, when carrying out measurements in the temperature range with devices containing LEDs, it is necessary to take into account the change in wavelength corresponding to the maximum of the emission spectrum. Thus, the shift in the emission maximum of the 940 nm IR LED used was approximately 32 nm, from 930 nm at a temperature of −25 °C to 962 nm at a temperature of +60 °C. It is important to note that the IR LEDs used for illumination were cooled simultaneously with the detector. Therefore, additional measurements are necessary to accurately assess this effect at a particular wavelength of IR illumination. As an alternative, measurements with an external IR illumination source with a narrow range of wavelengths that is independent of temperature are required. These can be IR bandpass filters, a monochromator or an IR laser diode with light guides.

## 4. Conclusions

The present work was focused on the study of the influence of IR illumination and temperature variation on the performance of quasi-hemispherical CdZnTe (CZT) detectors. The research contributions can be summarized as follows:

1. Two CZT planar detectors of dimensions 20 × 20 × 10 mm and two detectors of dimensions 15 × 15 × 7.5 mm were inspected with IR transmission microscopy and by the surface scanning of detectors with alpha particles. Irradiation with alpha particles was carried out from the cathode, which ensured the formation of output pulses owing to the collection of electrons. The inspection with IR microscopy did not reveal the presence of large inclusions or clusters of small inhomogeneities along the grain boundaries. The scanning of the surface of one of the planar detectors showed the presence of a noticeable nonuniformity in a certain region. Complex shapes of the rising edge in the pulses and reduced charge-collection efficiency from this region were discovered. Pulses from all other regions of this detector surface had linear rising edges, which indicated a uniform distribution of the electric field. IR illumination did not improve charge collection in all planar detectors. Analysis of the output signal waveforms allowed us to conclude that the space charge region was formed at their cathodes under the influence of the used IR illumination with wavelengths of 940 nm and 1050 nm.
2. The tested planar detectors were used to manufacture quasi-hemispherical detectors. Alpha spectra for different regions on the larger side of the cathode were measured using a digitizer. Amplitude to transit time distributions, amplitude spectra and transit time distributions for the different regions of the detector surface were obtained. All tested detectors exhibited poor charge collection from the peripheral regions of the detector, primarily from the corners. The output pulses from these regions had very long rising edges and reduced amplitudes compared to the pulses from the central region. A long rising edge is determined by a significantly lower electric field in the corner regions. In the detector, in the planar form of which the region of reduced charge-collection efficiency had been detected, the pulses from this and adjacent regions also had rather long rising edges.
3. IR illumination of the detectors with a wavelength of 940 nm at room temperature significantly improved charge collection from the peripheral regions. The rising edge of the output pulse became significantly shorter, and the amplitude increased noticeably. This result could be due to the redistribution of the electric field, an increase in the corner regions and a decrease in the center region. This redistribution, in turn, could be caused by the formation of a positive space charge near the cathode. If, in a planar detector, the formation of a space charge at the cathode did not lead to improved charge collection, then in a quasi-hemispherical detector, this effect gave a significant positive result. Improvement in characteristics when collecting charges from the central region also occurred, but noticeably less.
4. The behavior of the quasi-hemispherical detector with dimensions of 15 × 15 × 7.5 mm in the temperature range from −30 °C to +50 °C was studied. The amplitude spectra and the transit time distributions at the registration of alpha particles were obtained. The influence of IR illumination of different wavelengths on the detector parameters was studied. Charge collection from two regions in a corner and in the center of the detector surface was studied. In the corner region, polarization was discovered at a temperature of −30 °C, which was manifested by a significant deterioration in charge collection over time, i.e., the amplitude was decreasing, and the transit time was increasing. At higher temperatures, the detector performance remained stable over time. The use of IR illumination with a wavelength of 1050 nm caused depolarization, restoring the characteristics of the detector operating at −30 °C; no changes were observed over time at constant levels of illumination. With increasing temperature, the effect of IR illumination decreased and completely disappeared at temperatures above +40 °C. Conversely, the positive effect of IR illumination at a wavelength of 940 nm at a temperature of −30 °C was short-lived and only immediately after turning on the illumination. Over time, polarization appeared and manifested in a significant increase in transit time. With increasing temperature, the efficiency of the IR illumination increased, achieving a stable positive result in the temperature range from +10 °C to +50 °C. When collecting charges from the central region, the behavior of the detector was different. No polarization was observed either at −30 °C or at other temperatures; the obtained waveforms of output pulses, calculated amplitude spectra and transit time distributions did not change over time. IR illumination with both wavelengths of 940 and 1050 nm also did not have noticeable impacts on the waveforms and amplitudes of output pulses. Application of IR illumination at a wavelength of 1050 nm slightly decreased the transit time at −30 °C.

The main conclusions from the results obtained are as follows.

The deterioration of the characteristics of quasi-hemispherical detectors at low temperatures is determined primarily by a decrease in the charge-carrier collection from the peripheral regions of the detector. Since the electric field in these regions is initially already reduced, the formation of space charges in these regions is more probable than in the central region, where the electric field is higher. Correspondingly, IR illumination has primarily a positive effect on the charge collection from the peripheral areas of the detector.

To ensure optimal operating conditions for quasi-hemispherical detectors in a wide temperature range, it is necessary to use IR illumination with different wavelengths and intensities. It is possible to use for this an automatic system consisting of two or three LED IR sources controlled by a processor designed to control each of the sources depending on the operating temperature of the detector. Such a system based on microspectrometer µSPEC4000 [49] is already being developed and tested. Optimization of the proposed system requires a more detailed analysis of the results of the simultaneous use of IR illumination with different wavelengths at different temperatures.

Another method that can significantly improve charge collection in the detectors is to manufacture them with geometries approaching hemispherical. Removing the corner regions of a quasi-hemispherical detector, the presence of which is a significant factor of degradation, can significantly improve the performance of the detectors.

## Figures and Tables

**Table 1:** Positions of the maxima of the amplitude spectra and the maxima of the transit time distributions for all tested regions on the detector of the 20 × 20 × 10 mm.

Region No.	1	2	3	4	5	6	7	8	9	10	11	12	13
Amplitude peak maximum, V	0.14	0.16	0.16	0.16	0.15	0.15	0.16	0.15	0.08	0.15	0.15	0.14	0.14
Transit time distribution maximum, µs	7.4	3.0	2.5	2.4	2.4	3.0	2.9	4.4	8.0	2.8	3.0	3.3	3.4

**Table 2:** Positions of the maxima of the amplitude spectra and the maxima of the transit time distributions for all tested regions on the detector of the 20 × 20 × 10 mm surface with and without IR illumination at 940 nm.

Region No.	1	2	3	4	5	6	7	8	9	10	11	12	13
Amplitude peak maximum without IR illumination, V	0.08	0.13	0.11	0.14	0.11	0.14	0.01	0.11	0.09	0.15	0.15	0.14	0.15
Amplitude peak maximum with IR illumination, V	0.09	0.16	0.16	0.17	0.17	0.16	0.16	0.12	0.09	0.17	0.17	0.16	0.17
Transit time distribution maximum without IR illumination, µs	2.75	1.35	2.6	1.15	3	0.75	4.05	1.25	1.35	0.45	0.55	0.75	0.95
Transit time distribution maximum with IR illumination, µs	2.45	0.45	0.45	0.35	0.45	0.45	0.75	1.85	1.85	0.35	0.45	0.55	0.25

**Table 3:** Positions of the maxima of the amplitude spectra and the maxima of the transit time distributions for all tested regions on the detector of the 15 × 15 × 7.5 mm surface with and without IR illumination at 940 nm.

Region No.	1	2	3	4	5	6	7	8	9	10	11	12	13
Amplitude peak maximum without IR illumination, V	0.08	0.13	0.08	0.13	0.08	0.13	0.10	0.14	0.12	0.13	0.14	0.15	0.16
Amplitude peak maximum with IR illumination, V	0.15	0.16	0.16	0.16	0.16	0.16	0.15	0.16	0.16	0.16	0.16	0.16	0.16
Transit time distribution maximum without IR illumination, µs	0.81	0.39	1.29	0.45	0.93	0.45	0.65	0.33	0.39	0.39	0.39	0.35	0.35
Transit time distribution maximum with IR illumination, µs	0.45	0.39	0.39	0.45	0.45	0.39	0.45	0.45	0.39	0.39	0.39	0.34	0.34

## Data Availability

Data are contained within the article.
